# Gender-Related Effect in Oxygenation Dynamics by Using Far-Infrared Intervention with Near-Infrared Spectroscopy Measurement: A Gender Differences Controlled Trial

**DOI:** 10.1371/journal.pone.0135166

**Published:** 2015-11-10

**Authors:** Wei-Lung Kao, Chia-Wei Sun

**Affiliations:** Department of Photonics, National Chiao Tung University, Hsinchu, Taiwan, R.O.C.; National Yang-Ming University, TAIWAN

## Abstract

Many studies have indicated the microcirculation can directly respond to disease-related symptoms. However, the capacity of microcirculation would vary due to the gender differences. Near-infrared spectroscopy (NIRS) is a noninvasive technique to monitor tissue oxygenation dynamics. In this study, the far-infrared (FIR) source was used for physiological intervention of microcirculation. The experimental results show that the nature difference of oxygenation status exists between male and female during FIR irradiation. Therefore, we suggest the NIRS-based assessment should be calibrated with the gender-related effect for clinical diagnosis of peripheral arterial disease.

## Introduction

Vascular circulation capacity is a critical indicator of the state of the human body. Many methods have been proposed for evaluating vascular microcirculation in clinical diagnosis. The microcirculatory system is composed of small blood vessels that directly affect organ-related functions in oxygen and nutrient delivery [1]. Therefore, microcirculatory function directly indicates the status of tissue oxygenation. Variation in microcirculatory function is due to innate physiological differences between men and women. Hormone-dependent sex differences exist in vascular function [2]. Estrogen causes vasodilatation by both rapidly increasing nitric oxide (NO) production and inducing nitric oxide synthases (NOS) genes. Blood pressure is lower in male adolescents than in premenopausal female adolescents of the same age and rises following the onset of menopause [3]. Both vasodilatation and blood pressure are affected by fluctuating levels of circulating estrogen during the menstrual cycle and pregnancy [4,5].

Currently, the clinical assessment of cardiovascular adequacy typically involves noninvasive hemodynamic monitoring based on near-infrared spectroscopy (NIRS), which can provide an evaluation of activation-related tissue oxygenation changes. Since NIRS methods can provide the spatial distribution of tissue oxygenation in real time, they are an effective tool for estimating microcirculation in tissue. Thus, NIRS can be used to assess the oxidative metabolism of skeletal muscle. To avoid the use of an exogenous tracer, one study [6] proposed analyzing the abrupt changes of oxyhemoglobin (HbO2) and deoxyhemoglobin (Hb) after vessel occlusion.

Vessel occlusion tests such as the venous occlusion test and arterial occlusion test (AOT) are common physiological intervention methods for assessing microcirculatory function. However, the drawbacks of vessel occlusion tests including skin contact, discomfort, and microcirculation block may let the method not be used in some diseases. Thus, we proposed far-infrared (FIR) illumination as a novel physiological intervention method. Infrared radiation transfers energy in the form of heat, which is sensed by skin thermoreceptors when they are illuminated [7]. FIR therapy (3–25 μm) is effective for increasing skin microcirculation [8] and has been applied to treat many vascular-system-related disorders [9,10]. In our previous study, we showed that a relationship exists between AOTs and FIR illumination tests for detecting tissue oxygenation through NIRS assessment. The results of that study indicated that FIR illumination tests might be a suitable physiological intervention for replacing AOTs [11].

A previous study proposed using NIRS measurements for evaluating patients with peripheral arterial disease (PAD) [12]. However, intrinsic differences in oxygenation dynamics were not observed between men and women using NIRS measurements. Therefore, we used NIRS measurements to record the hemodynamic changes that occurred during an FIR illumination test and separated them according to sex to analyze the characteristics discussed in this paper.

## Materials and Methods

All participants provided written informed consent. This study was conducted in accordance with the latest version of the Declaration of Helsinki and approved by the institutional review board (IRB) committee at National Chiao Tung University, Taiwan. Participation in the experiment was voluntary, and the participants were required to sign a consent form in accordance with approval of the IRB.

### Healthy participants

Twenty-five healthy volunteers (14 men, 11 women) were recruited in the study. The average age of the subject population was 22.08 (± 1.97 years), and all subjects were neither overweight (body mass index, BMI ≥ 24) nor underweight (BMI ≤ 18.5). For 3 hours before the experiment, the subjects were asked to refrain from drinking caffeinated beverages and performing exercise. Particular emphasis was placed on not disturbing the women’s menstrual cycle.

### Far Infrared Therapy Unit (TY-101F)

The FIR illumination test was performed using an FIR emitter (TY-101N, WS Far Infrared Medical Technology Co. Ltd., Taipei, Taiwan). The emitter has three intensity settings: low, medium, and high. The wavelength of the light generated from the electrified ceramic plates ranged between 5 and 12 mm, with a peak at 8.2 mm. The emitter was placed 20 cm above the forearm to enable the forearm skin temperature to be increased gradually. At a distance of 20 cm and the high intensity setting, the power density received by the irradiated body is 20 mW/cm^2^. Before FIR illumination was applied, data over a 2-min stabilization period were recorded as the baseline.

### Near-infrared spectroscopy measurements

Static and dynamic measurements were obtained using a continuous-wave system (PortaLite, Artinis, Medical System, Zetten, the Netherlands). The system used in this study was a two-wavelength (760 and 850 nm) continuous-wave system with a sampling rate of 50 Hz that simultaneously used the modified Beer—Lambert [Eq (1)] and spatially resolved spectroscopy methods to measure the HbO_2_ and Hb in the region of interest.

$$OD_{\lambda }\;=\;log\frac{I_{0}}{I}\;=\;\varepsilon_{\lambda }\cdot c\cdot L\cdot B_{\lambda }+OD_{R,\lambda }$$ (1)

In Eq (1), *OD*_*λ*_ is a dimensionless factor known as the optical density of the medium; *I*_0_ is the incident radiation; *I* is the transmitted radiation, ɛ_λ_ (mM^-1^/cm^-1^) is the extinction coefficient of the chromophore; *c* (mM^-1^) is the concentration of the chromophore; *L* (cm) is the distance between the light entry and exit points; λ (nm) is the wavelength; *B*_λ_ is the differential pathlength factor, which is a dimensionless pathlength correction factor; and *OD*_R,*λ*_ represents the oxygen-independent light losses attributable to scattering in the tissue. Assuming that *OD*_R_, remains constant during a measurement, we converted an optical density change into a concentration change as follows:

$$\Delta OD_{\lambda }\;=\;OD_{final}-OD_{initial}\;=\;\text{log}\frac{I_{initial}}{I_{fianl}}\;=\;\varepsilon_{\lambda }\cdot \Delta c\cdot L\cdot B_{\lambda }\;\to \Delta c\;=\;\frac{\Delta OD_{\lambda }}{\varepsilon_{\lambda }\cdot L\cdot B_{\lambda }}$$ (2)

where *OD*_initial_ and *OD*_fianl_ are the initial and instantaneous values of the optical density of the medium, respectively; *I*_initial_ and *I*_final_ are initial and instantaneous values of radiation, respectively; and Δ*OD0*_*λ*_ and Δ*c* are the changes in the relative amount before and after the test, respectively. This equation is valid for a medium with one chromophore. In biological tissue, two oxygenation-dependent chromophores are present: HbO_2_ and Hb. Eq (2) was rewritten as a dual linear equation as follows:

$$\Delta OD_{\lambda }=(\varepsilon_{\lambda ,HbO2}\cdot \Delta \left[HbO_{2}\right]+\varepsilon_{\lambda ,Hb}\cdot \Delta \left[Hb\right])\cdot L\cdot B_{\lambda }$$ (3)

Two wavelengths (760 and 850 nm) were used to solve Δ[HbO_2_] and Δ[Hb]. Eq (3) was rewritten as dual linear simultaneous equations as follows:

$$\left\{ \begin{array}{c} \Delta OD_{760}=\left(\varepsilon_{760,HbO2}\cdot \Delta \left[HbO2\right]+\varepsilon_{760,Hb}\cdot \Delta \left[Hb\right]\right)\cdot L\cdot B_{760}\\ \Delta OD_{850}=\left(\varepsilon_{850,HbO2}\cdot \Delta \left[HbO2\right]+\varepsilon_{850,Hb}\cdot \Delta \left[Hb\right]\right)\cdot L\cdot B_{850} \end{array}\right.$$ (4)

The concentration of HbO_2_ and Hb can be calculated using Eq (4), where ɛ_760,HbO2_ = 0.586 mM^-1^·cm^-1^, ɛ_760,Hb_ = 1.548 mM^-1^·cm^-1^, ɛ_850,HbO2_ = 1.058 mM^-1^·cm^-1^, ɛ_850,Hb_ = 0.691 mM^-1^·cm^-1^, L = 3.5 cm, and both B_760_ and B_850_ = 4.

### Measurement protocol

**Fig 1:**
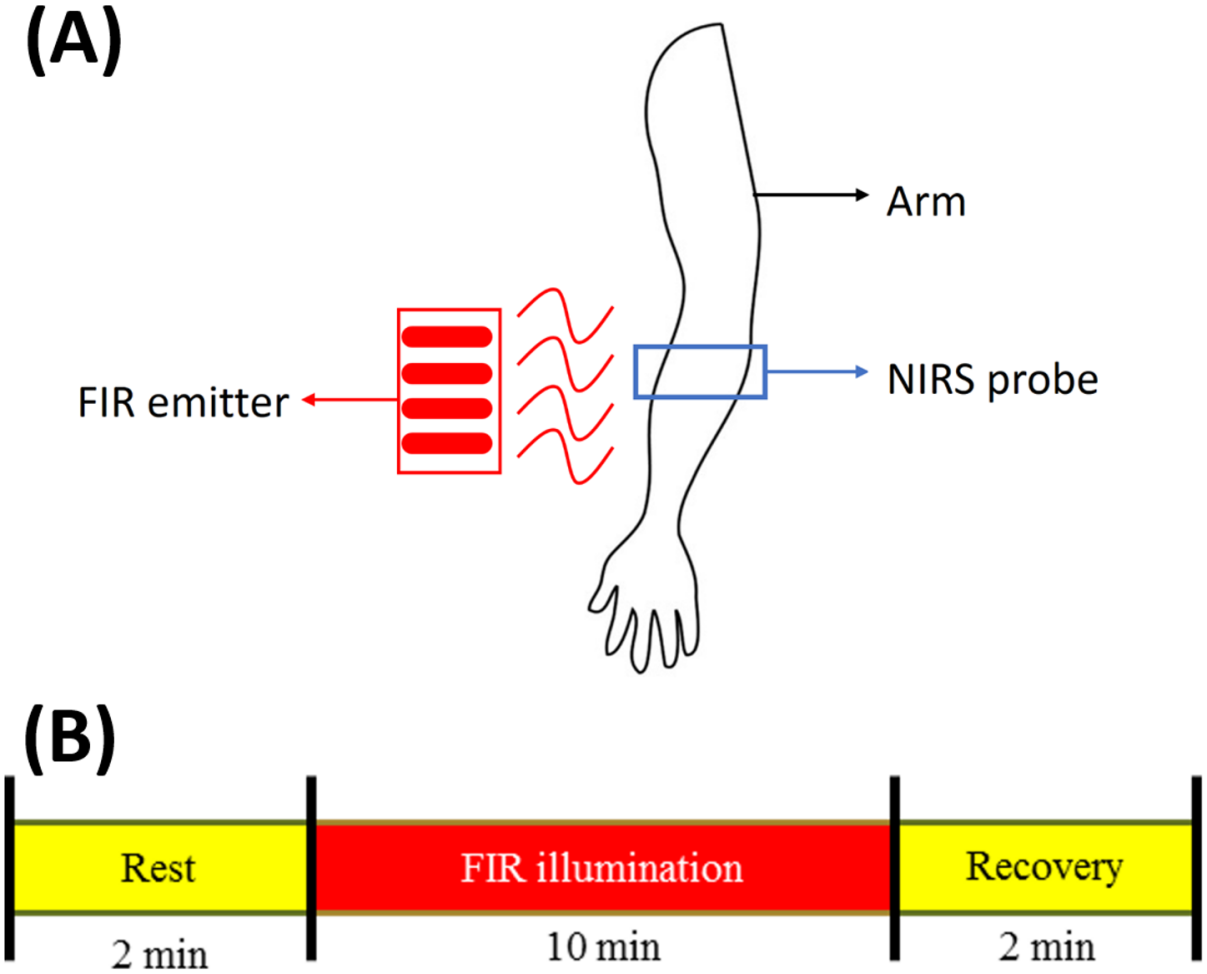
Measurement setup (A)and measurement protocol (B).

The study was designed as a prospective case-controlled clinical investigation and informed consent was obtained from 25 healthy volunteers. All subjects rested in a recumbent position in a quiet environment with a room temperature of 27°C. No movement was allowed throughout the NIRS monitoring process. An NIRS probe was placed on the skin of the brachioradialis to assess the tissue oxygenation status by using near-infrared optical detection (Fig 1A). The temporal tracings of oxygenation signals were obtained under FIR illumination. The NIRS measurement was 14 min in duration and conducted according to the following three steps: (1) rest; (2) FIR illumination; and (3) recovery (Fig 1B). The participants first rested for 2 min as a stabilization period, during which baseline measurements were taken. After 10 min of FIR illumination, the FIR emitter was turned off and the oxygenation recovery signals were monitored for 2 min.

### Statistics and analysis

The data from the NIRS measurements including Δ[HbO_2_] and Δ[Hb] changed over a 14-min period. The trend was obtained by the method of arithmetic mean. All the subjects at each time would get each arithmetic mean, and the slope was obtained by performing linear regression. In addition, we calculated the standard deviation to ensure that the data were trustworthy. All data were normalized before additional comparison to reduce the influence of individual differences and provide a common scale for the variables. We regard one subject as a matrix and normalized the matrix. After the data were all normalized, the mean and standard deviation were obtained.

## Results and Discussion

### All subjects

**Fig 2:**
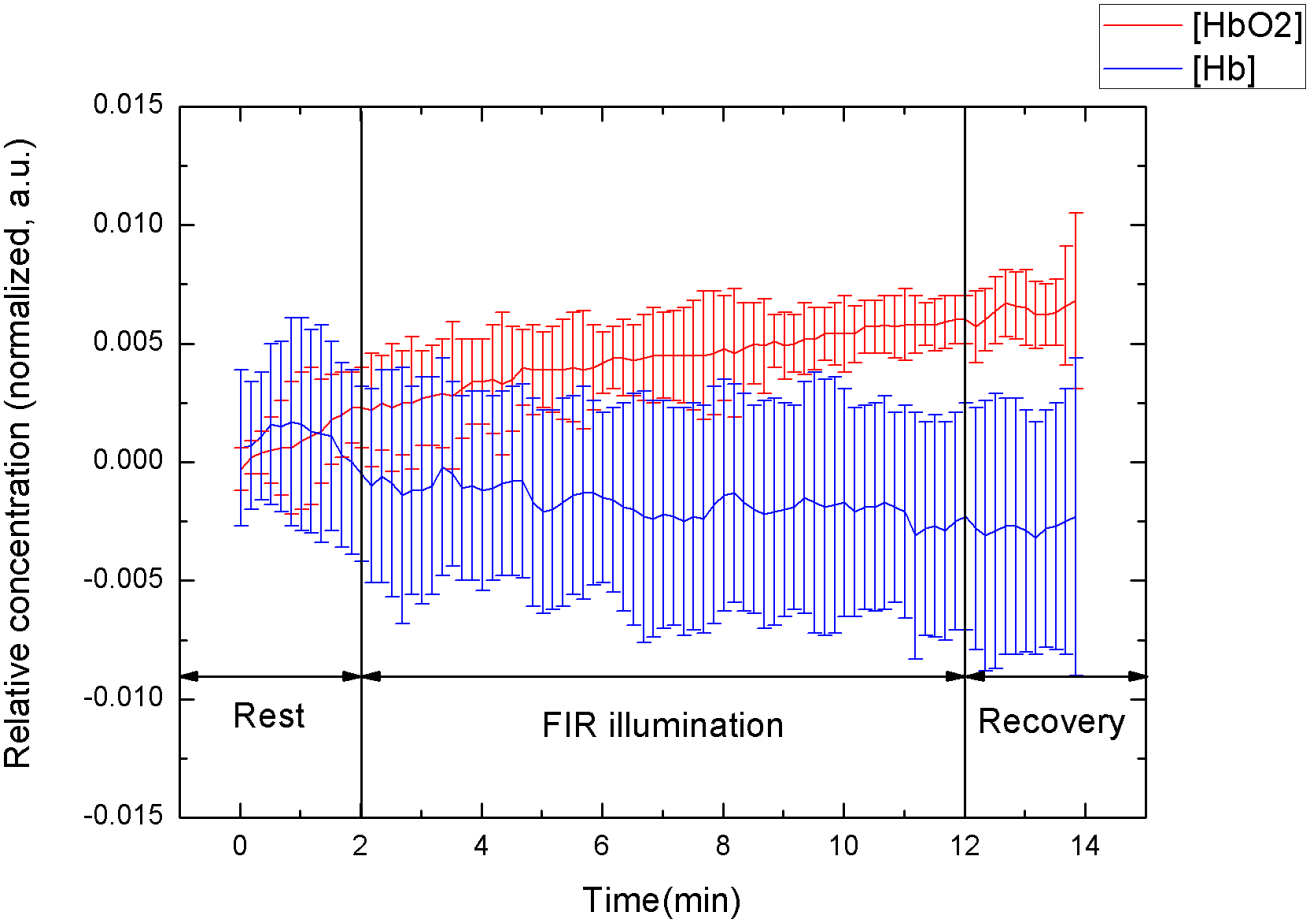
Temporal tracings of signal response to FIR illumination of all subjects.

The mean of all subjects can be used to determine the slope to indicate the increase (or decrease) in temperature per minute at the FIR illumination step. The slope of Δ[HbO_2_] and Δ[Hb] among all subjects was 0.29 ± 0.20 μM·min^-1^ and -0.03 ± 0.09 μM·min^-1^, respectively. Fig 2 shows the mean values of the temporal tracings of tissue oxygenation in all subjects. During FIR illumination, the forearm temperature increased steadily. FIR illumination can improve microcirculation through its thermal effects. The concentration of HbO_2_ increased and the concentration of Hb decreased. At the recovery step, Δ[HbO_2_] continued to increase slightly because of nonthermal effects [13]. The results prove that NIRS measurement can be performed to directly monitor changes in microcirculation.

### Stratification of subjects according to sex

**Fig 3:**
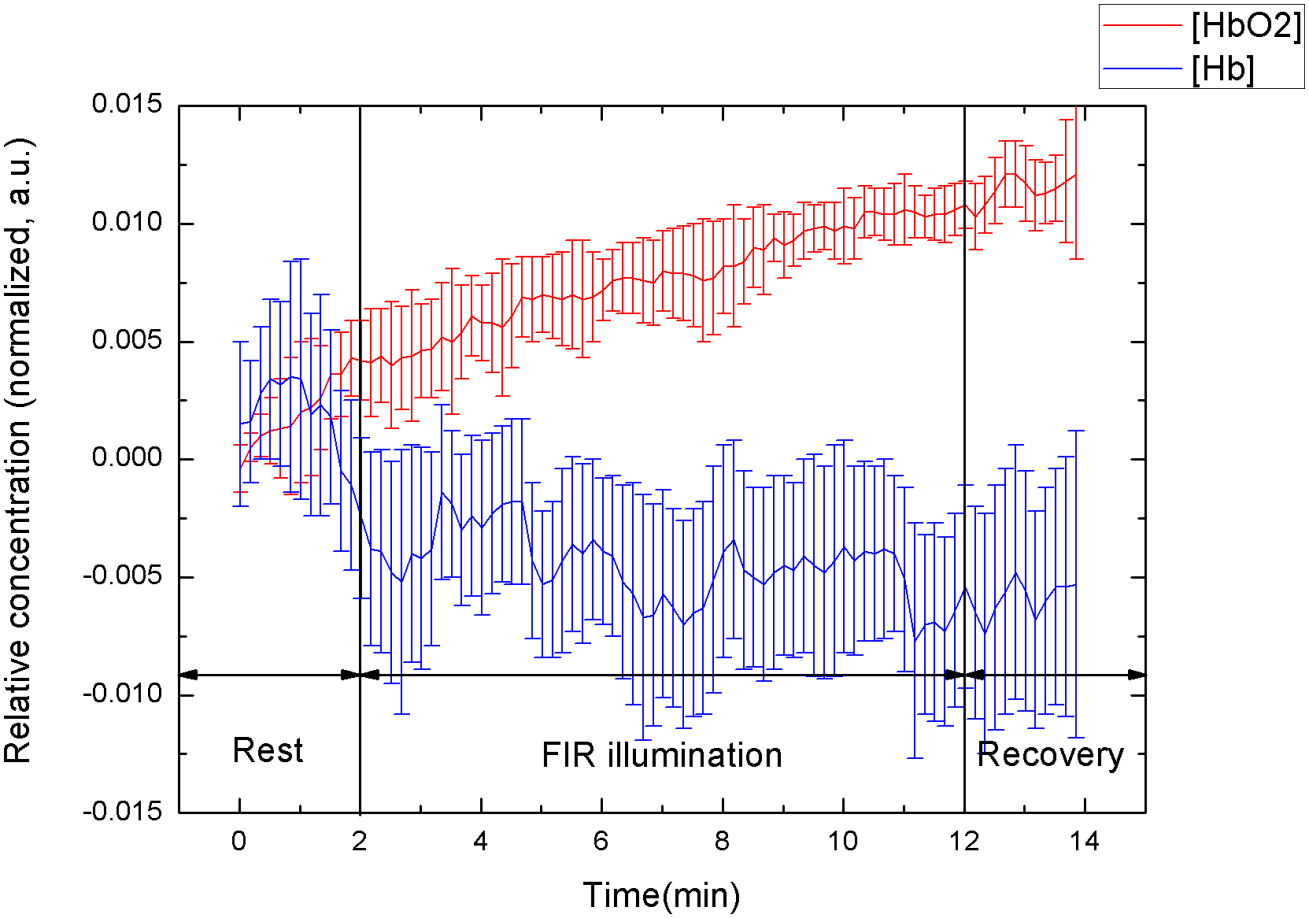
Temporal tracings of signal response to FIR illumination of 14 male.

**Fig 4:**
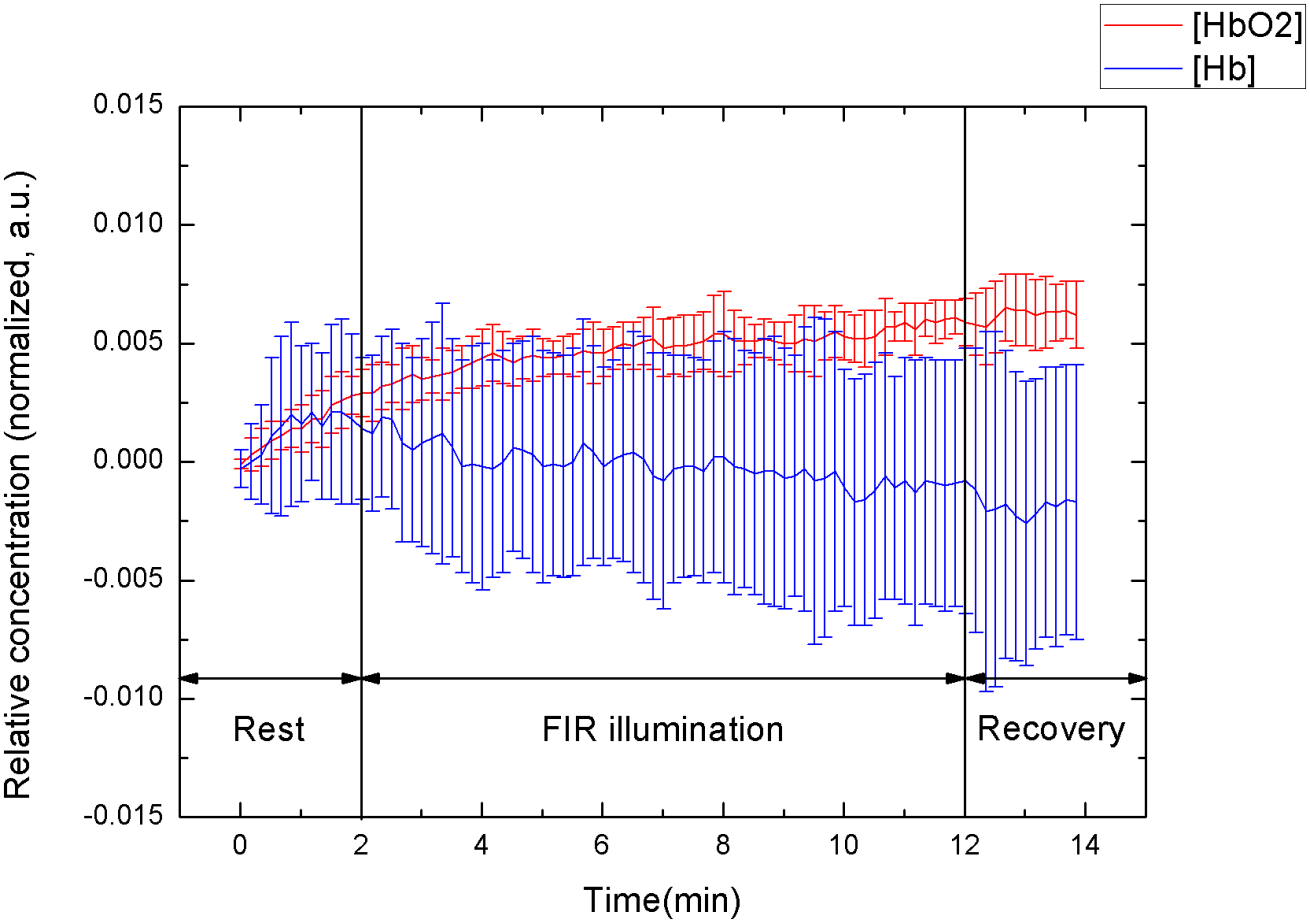
Temporal tracings of signal response to FIR illumination of 11 female.

The Δ[HbO_2_] and Δ[Hb] slopes of the 14 male subjects were 0.37 ± 0.22 μM·min^-1^ and -0.03 ± 0.10 μM·min^-1^, respectively, whereas those of the 11 female subjects were 0.19 ± 0.15 μM∙min^-1^ and -0.03 ± 0.08 μM∙min^-1^, respectively. Figs 3 and 4 show the mean values of the temporal tracings of tissue oxygenation in the 14 men and 11 women, respectively.

The men’s Δ[HbO_2_] slope was steeper than that of the women (0.37 > 0.19). Although the Δ[Hb] slopes were relatively similar, the effect of FIR on the men was stronger than that on the women. Figs 3 and 4 show the sex-based differences in the effects of FIR illumination on the biological parameters. Microcirculation changed more in the men than in the women. During FIR illumination, the rate of changes in oxyhemoglobin in the men was approximately twofold higher that in the women. Following FIR illumination, ΔHbO_2_ in the men continued to increase, whereas that in the women was remained constant. Previous studies have shown that there are innate differences between the sexes [2–5]. FIR illumination causes biothermal effects that promote regional vasodilation. Estrogen can regulate vasodilatation [2]; consequently, vasodilation by FIR illumination was not apparent in the female subjects. With less estrogen and relatively stronger skeletal muscle, the male subjects exhibited a markedly greater increase in Δ[HbO_2_].

## Conclusion

In conclusion, we used NIRS to detect the temporal tracings of tissue oxygenation by performing an FIR illumination test. The changes in microcirculation were successfully monitored using NIRS. Moreover, we separated the subjects according to sex and observed sex-specific differences in the NIRS measurements. Microcirculation differs among patients and between healthy and unhealthy people. However, no study has focused on using NIRS measurements to examine sex-specific differences in microcirculation. Collecting an adequate database of sex-based differences, as well as differences in physiological parameters such has BMI and age, may facilitate establishing a normalized standard. The finding that microcirculatory function varies according to sex is in agreement with the findings of previous studies [2–5]. Caution must be taken when interpreting our findings because the results of this study were affected by differences in optical properties; consequently, we cannot confirm what the main factor is. Many physiological parameters, such as the ratio of skeletal muscle and thickness of subcutaneous fat, vary according to sex. Estrogen is the most consistently identified source of these differences in many previous studies. FIR illumination for detecting microcirculation is nonoppressive and carries no risk; therefore, it is advantageous for intensive care units. Future studies should first consider collecting additional data. In addition, NIRS measurements with FIR illumination have high potential for application in clinical practice. With the advantages of noninvasiveness and real-time results, the method is suitable for managing many diseases, such as PAD. The results of this study show that NIRS measurements with FIR illumination can be used to monitor changes of microcirculation in real time.

## Supporting Information

S1 File (XLSX)

S2 File (XLSX)

S3 File (XLSX)

S4 File (XLSX)

S5 File (XLSX)

S6 File (XLSX)

S7 File (XLSX)

S8 File (XLSX)

S9 File (XLSX)

S10 File (XLSX)

S11 File (XLSX)

S12 File (XLSX)

S13 File (XLSX)

S14 File (XLSX)

S15 File (XLSX)

S16 File (XLSX)

S17 File (XLSX)

S18 File (XLSX)

S19 File (XLSX)

S20 File (XLSX)

S21 File (XLSX)

S22 File (XLSX)

S23 File (XLSX)

S24 File (XLSX)

S25 File (XLSX)
